# Risk of admission to hospital for self-harm after admission to hospital for COVID-19: French nationwide longitudinal study

**DOI:** 10.1192/bjo.2024.786

**Published:** 2024-12-05

**Authors:** Philippe Pirard, Valentina Decio, Baptiste Pignon, Olivier Bouaziz, Vittorio Perduca, Viviane Kovess-Masfety, Emmanuelle Corruble, Francis Chin, Pierre A. Geoffroy, Yann Le Strat, Jonathan Messika, Nolwenn Regnault, Sarah Tebeka

**Affiliations:** Non Communicable Diseases and Trauma Division, Santé publique France, Paris, France; Département Médico Universitaire (DMU) – Innovation en santé Mentale, Psychiatrie et AddiCTologie, Hôpitaux Universitaires « H. Mondor », Assistance Publique hôpitaux de Paris (AP-HP), Université Paris-Est-Créteil (UPEC), Mondor Biomedical Research Institute (IMRB - Inserm), Translational Neuropsychiatry, Fondation FondaMental, Créteil, France; Université Paris Cité, CNRS, MAP5, Paris, France; Non Communicable Diseases and Trauma Division, Santé publique France, Paris, France; and Laboratoire de Psychopathologie et Processus de Santé, Université Paris Cité, Paris, France; CESP, MOODS Team, INSERM UMR 1018, Faculté de Médecine, Université Paris-Saclay, Paris, France; and Service Hospitalo-Universitaire de Psychiatrie de Bicêtre, Hôpitaux Universitaires Paris-Saclay, AP–HP, Hôpital de Bicêtre, Paris, France; Data Science Division, Santé publique France, Paris, France; Département de Psychiatrie et d'Addictologie, AP-HP, Centre ChronoS, Groupe Hospitalier Universitaire (GHU) Paris Nord, DMU Neurosciences, Hospital Bichat - Claude Bernard, Paris, France; (GHU) Paris - Psychiatry & Neurosciences, Paris, France; and Université de Paris, NeuroDiderot, INSERM, FHU I2-D2, Paris, France; Service de Pneumologie B et Transplantation Pulmonaire, Hôpital Bichat-Claude Bernard, AP-HP, Nord-Université Paris Cité, Paris, France; and Physiopathology and Epidemiology of Respiratory Diseases, UMR1152 INSERM and Université de Paris, Paris, France; Non Communicable Diseases and Trauma Division, Santé publique France, Paris, France; Department of Psychiatry, AP-HP, Louis Mourier Hospital, Paris, France; and INSERM Team 1 – UMR1266, Institute of Psychiatry and Neurosciences, Université de Paris, Paris, France

**Keywords:** COVID-19, epidemiology, admission to hospital, suicide attempts, psychiatric disorders

## Abstract

**Background:**

Assessing the risk of subsequent self-harm after hospitalisation for COVID-19 is critical for mental health care planning during and after the pandemic.

**Aims:**

This study aims to compare the risk of admission to hospital for self-harm within 12 months following a COVID-19 hospitalisation during the first half of 2020, with the risk following hospitalisations for other reasons.

**Method:**

Using the French administrative healthcare database, logistic regression models were employed to analyse data from patients admitted to hospitals in metropolitan France between January and June 2020. The analysis included adjustments for sociodemographic factors, psychiatric history and the level of care received during the initial hospital stay.

**Results:**

Of the 96 313 patients hospitalised for COVID-19, 336 (0.35%) were subsequently admitted for self-harm within 12 months, compared to 20 135 (0.72%) of 2 797 775 patients admitted for other reasons. This difference remained significant after adjusting for sociodemographic factors (adjusted odds ratio (aOR) = 0.66, 95% CI: 0.59–0.73), psychiatric disorder history (aOR = 0.65, 95% CI: 0.58–0.73) and the level of care received during the initial hospital stay (aOR = 0.70, 95% CI: 0.63–0.78). History of psychiatric disorders and intensive care were strongly correlated with increased risk, while older age was inversely associated with self-harm admissions.

**Conclusions:**

Hospitalisation for COVID-19 during the early pandemic was linked to a lower risk of subsequent self-harm than hospitalisation for other reasons. Clinicians should consider psychiatric history and intensive care factors in evaluating the risk of future suicide.

## A need for knowledge about the links between COVID-19 and suicide risk

The burden of the numerous admissions to hospital for COVID-19 on suicide risk is a public health issue that still deserves investigations.^1^ Studies conducted during the COVID-19 pandemic report the negative impact of the crisis on the mental health of general populations,^2^ suggesting that this risk of suicide or of self-harm was substantial. This impact would appear to be stronger in persons infected by COVID-19.^3^ In addition, admissions to an intensive care unit (ICU) for severe COVID-19 infection have a particularly strong impact on mental health and psychiatric disorders.^4,5^ People with psychiatric disorders classically present a higher risk of suicide and self-harm.^6^ Moreover, the unprecedented COVID-19 pandemic and its associated political and impacts may have affected suicidality in many ways. Examples include social distancing and lockdowns, which contributed to a feeling of isolation and loneliness,^7,8^ and the crisis that led to unemployment and loss of income in many sectors.^8,9^ This might particularly concern people affected by COVID-19, which can contribute to suicidal ideation by increasing hopelessness and social isolation, or through the psychiatric effects of the illness.^10^ Some authors have raised concerns over a possible suicide epidemic and a potential ‘suicide and COVID-19 double pandemic’.^8^ As far as we know, no study conducted to date on medical consultations or admissions to hospital for self-harm in relation to the COVID-19 crisis has clearly confirmed this double epidemic.^11^ Studies of primary care records, emergency department visits and admissions to hospital for self-harm or attempted suicide showed lower rates of self-harm or suicide attempts after each country's first lockdown period^12,13^ or did not find any significant differences.^14^ French data even show lower death rates in 2020 than in previous years, including deaths by suicide, particularly during lockdowns.^15^

## Does COVID-19 particularly affect the risk of suicide attempts after hospitalisation?

Most of the published data on suicidality concern the entire population affected by the pandemic. Specific data on people who were admitted to hospital for COVID-19 infection are much rarer. It has been established that the probability of self-harm increases after admission to hospital irrespective of the reason.^16^ It has also been suggested that persons with a history of COVID-19 are more likely to present suicide ideation than others.^17^ In a retrospective web survey of a large sample of US students conducted between September and December 2020, DeVylder et al^10^ showed a higher prevalence of self-harm in the past year among those who had been admitted to hospital for COVID-19, compared with those who were not infected by COVID-19. Thus, in this unique context of a major health crisis, the available data are insufficient to have a better understanding of the impact of admission to hospital for COVID 19 on the risk of subsequent self-harm. Given these elements, the question arises whether admission to hospital owing to COVID-19 specifically affects the subsequent risk of admission to hospital for self-harm, compared to admission to hospital for other reasons.

A recent study found that among the 2 894 088 adults admitted to hospital during the first half of 2020, in France, the proportion of patients subsequently admitted to hospital for a psychiatric disorder within 12 months of discharge was significantly higher for those initially admitted to hospital for COVID-19 (11.09% *v.* 9.24% for other reasons, odds ratio = 1.20, 95% CI: 1.18–1.23, *P* < 0.001).^4^ Using the same data-set, this study aimed to compare the frequency of admission to hospital for self-harm within 12 months following discharge after admission to hospital owing to COVID-19 versus admission to hospital for other reasons in the French adult population during the first half of 2020.

## Method

### Data sources

We employed data from the French administrative healthcare database – Système National des Données de Santé (SNDS [National Health Data System]) – encompassing nearly the entire French population. The SNDS comprises pseudonymised databases that include mandatory health insurance data, that is, reimbursement data, specifically those derived from the processing of healthcare reimbursement requests and data from healthcare institutions (Programme de Médicalisation des Systèmes d'Information (PMSI [French National Hospital Discharge Database])).

In our study, we specifically used the PMSI, which furnishes details regarding admissions and discharges for stays in hospital across both public and private structures. Each hospital stay was coded using the International Statistical Classification of the Diseases (ICD-10^18^) for medical diagnoses. A distinct patient identification number was assigned for various admissions involving the same patient.^19^ Additional details concerning the PMSI can be found elsewhere.^4,20,21^

To identify psychiatric history in the 5 years before the study period, we relied from the medical algorithms based on the SNDS data ‘Diseases and Expense Mapping’ (for details, see section ‘History of psychiatric disorder’ in this article).

### Study design and participants

This retrospective longitudinal study aimed to assess the risk of admission to hospital for self-harm during the 12 months following hospital discharge for COVID-19 or for another reason from a medical, surgical or obstetrics ward. The study focused on adults aged 18 years or older, and the timeframe for data collection was from 1 January 2020 to 30 June 2020 in metropolitan France.^4,20,21^

A reference hospital stay was selected for each individual. In cases of multiple admissions to hospital during the study period, the stay involving COVID-19 was considered the reference if at least one occurred. For patients with multiple admissions to hospital (for both COVID-19 or not), the highest level of clinical care provided determined the reference stay.^4^ The identification of the COVID-19-related admissions to hospital was carried out following coding guidelines from the Technical Agency for Information on Hospitalisation, which provides expertise on the collection and analysis of data on hospital activity. Patients were considered to have been admitted to hospital for COVID-19 if they had a (primary, related or associated) diagnosis with ICD-10 diagnosis codes U07.1, U07.10, U07.11, U07.12, U07.14 and U07.15.

### Outcome

The outcome was admission to hospital for self-harm in the 12 months following discharge for the initial (i.e. COVID-19 or another reason) hospital stay. We looked for inpatient and outpatient admissions that presented the ICD-10 codes for Intentional Self-Harm (X60–X84) registered as associated diagnoses in discharge reports. As these PMSI data could not provide information on the intention to die,^22^ we used this indicator, which includes self-harm, as an indicator of the latter.

### Variables of interest

#### Sociodemographic characteristics

Demographic variables available in the PMSI were age, gender and region of residence. Age was divided into four groups: 18–39 years, 40–59 years, 60–74 years and 75+ years. Socioeconomic status was measured using the French Deprivation Index (Fdep) in 2016, developed by the Centre d'Épidémiologie sur les Causes Médicales de Décès (CépiDc [Epidemiology Center on Medical Causes of Death]), which takes into account median household income, the percentage of high school graduates, the percentage of manual workers and the unemployment rate in the individual's city of residence.

#### History of psychiatric disorder

Using the CépiDc database, history of psychiatric disorder in the 5 years before the study period was defined by one of the following conditions^4^ for a given year, if one of the following was found in the SNDS for that year:

- declaration by a healthcare professional that the patient had a psychiatric disorder officially recognised as a long-term disease (in France, healthcare cover for such diseases is fully reimbursed);
- admission(s) for a psychiatric disorder in a psychiatric and/or non-psychiatric hospital during the previous 2 years (*n* to *n* − 1);
- admission(s) for a psychiatric disorder in a psychiatric and/or non-psychiatric hospital during the previous 5 years (*n* to *n* − 4), and prescription of specific psychotropic drugs on at least three different occasions during the current year *n*.

#### Characteristics of initial hospital stay

To characterise the level of intensity of care received during the initial hospital stay (i.e. for COVID-19 or for another reason), we considered its duration (median days) and three levels of clinical care received. These three levels were defined according to care provided in general for different degrees of COVID-19 severity (see details in Decio et al^4^).

### Statistical analyses

We first described our study population according to the reason (i.e. COVID-19 versus other reason) for their initial hospital stay. We then used logistic regression models to estimate and compare the risks of admission to hospital for self-harm in individuals initially admitted for COVID-19 and in those initially admitted for another reason.

Four nested models were subsequently performed for each outcome:

- Model 1: univariate model describing the unadjusted association between the main outcome (i.e. admission to hospital for self-harm, yes/no) and the reason for initial admission to hospital;
- Model 2: Model 1 adjusted for sociodemographic covariates;
- Model 3: Model 2 adjusted for psychiatric disorder history;
- Model 4: Model 3 adjusted for the characteristics of the initial hospital stay (median duration and level of clinical care received).

We conducted stratified analyses as follows:

- Model 5: Model 4 with no adjustment for gender but stratified according to this variable;
- Model 6: Model 4 with no adjustment for age but stratified according to the four different age categories;
- Model 7: Model 4 with no adjustment for intensity of clinical care but stratified according to the three different levels of this variable.

We have also proposed different sensitivity analyses to validate our results:

- we considered a shorter period for the initial admission to hospital (COVID-19 or other reasons) centred on the ‘COVID-19’ period, that is, from 16 March to 30 June 2020;
- in the control group (admission to hospital for a reason other than COVID-19), we excluded initial admissions to hospital for psychiatric reasons (i.e. 2.62% of the sample, see Decio et al^4^).

Statistical analyses were performed using SAS software, version 7.1 (Cary, NC, USA).

### Ethical considerations

The SNDS comprises a set of strictly pseudonymised and protected databases without any possibility to identify people. By law, Santé publique [Public Health] France has permanent regulatory access to SNDS data for the performance of its missions (article L.1461-3 and R1461-13) and following of the French public health code. Access to individual data in these systems for research purposes is only possible in the SNDS hub and the data cannot be extracted and shared. This access is not subject to the prior opinion of an ethics committee, nor to the authorisation of the Commission Nationale de l'Informatique et des Libertés (CNIL [National Commission on Information Technology and Civil Liberties]). Ethics approval and written informed consent were not relevant for this research on already existing data and were not required.

## Results

### Cohort description

Between 1 January 2020 and 30 June 2020, 2 894 088 individuals were admitted at least once to medical (including ICU), surgical and obstetrics wards in metropolitan France. Of these, 96 313 (3.32%) were admitted to hospital for COVID-19 and 2 797 775 (96.68%) for other reasons. The cohort's characteristics are presented by Decio et al (Table 1).^4^

**Table 1:** Comparison (in bold when statistically significant) of patients admitted to hospital for COVID-19 and those admitted to hospital for another reason among the whole sample and the sample of patients that self-harmed

Characteristic	Patients admitted during the first half of 2020				Patients admitted for self-harm in the 12 months following discharge after admission during the first half of 2020			
	Total (*n* = 2 894 088)	Admissions for COVID-19 (*n* = 96 313)	Admissions for another reason (*n* = 2 797 775)	*P-*value	Total (*n* = 20 471)	Admissions for COVID-19 (*n* = 336)	Admissions for another reason (*n* = 20 135)	*P-*value
Gender				**<0**.**0001**				0.5807
Male	1 270 314 (43.89)	50 699 (52.64)	1 219 615 (43.59)		9140 (4465)	155 (46.13)	8985 (44.62)	
Female	1 623 774 (56.11)	45 614 (47.36)	1 578 160 (56.41)		11 331 (55.35)	181 (53.87)	11 150 (55.38)	
Age (years)				**<0**.**0001**				**<0**.**0001**
18–39	669 227 (23.12)	7940 (8.24)	661 287 (23.64)		7924 (38.71)	66 (19.64)	7858 (39.03)	
40–59	612 000 (21.15)	20 701 (21.49)	591 299 (21.13)		8038 (39.27)	121 (36.01)	7917 (39.32)	
60–74	750 466 (25.93)	26 436 (27.45)	724 030 (25.88)		2701 (13.19)	57 (16.96)	2644 (13.13)	
75+	862 395 (29.8)	41 236 (42.81)	821 159 (29.35)		1808 (8.83)	92 (27.38)	1716 (8.52)	
Region				**<0**.**0001**				**<0**.**0001**
Île-de-France	435 139 (15.04)	33 999 (35.30)	401 140 (14.34)		2129 (10.40)	100 (29.76)	2029 (10.08)	
Centre-Val de Loire	115 194 (3.98)	2936 (3.05)	112 258 (4.01)		779 (3.81)	16 (4.76)	763 (3.79)	
Bourgogne-Franche-Comté	138 196 (4.78)	4872 (5.06)	133 324 (4.77)		1015 (4.96)	21 (6.25)	994 (4.94)	
Normandy	157 841 (5.45)	2736 (2.84)	155 105 (5.54)		1477 (7.22)	12 (3.75)	1465 (7.28)	
Hauts-de-France	287 055 (9.92)	9754 (10.13)	277 301 (9.91)		3300 (16.12)	46 (13.69)	3254 (16.16)	
Grand Est	251 670 (8.70)	14 752 (15.32)	236 918 (8.47)		1723 (8.42)	29 (8.63)	1694 (8.41)	
Pays de la Loire	165 845 (5.73)	2890 (3)	162 955 (5.82)		1259 (6.15)	18 (5.36)	1241 (6.16)	
Brittany	156 072 (5.39)	1487 (1.54)	154 585 (5.53)		1534 (7.49)	6 (1.79)	1528 (7.59)	
Nouvelle-Aquitaine	295 320 (10.20)	3169 (3.29)	292 151 (10.44)		2057 (10.05)	14 (4.17)	2043 (10.15)	
Occitanie	273 117 (9.44)	3699 (3.84)	269 418 (9.63)		1580 (7.72)	8 (2.38)	1572 (7.81)	
Auvergne-Rhône-Alpes	357 598 (12.36)	10 237 (10.63)	347 361 (12.42)		2317 (11.32)	48 (14.29)	2269 (11.27)	
Provence-Alpes-C. d'Azur	245 258 (8.47)	5523 (5.73)	239 735 (8.57)		1261 (6.16)	18 (5.36)	1243 (6.17)	
Corsica	15 783 (0.55)	259 (0.27)	15 524 (0.55)		40 (0.20)	0 (0.0)	40 (0.20)	
Social deprivation index (quintiles)				**<0.0001**				**<0.0001**
1 (least deprived)	476 939 (16.74)	22 591 (23.76)	454 348 (16.5)		2635 (13.05)	74 (22.36)	2561 (12.89)	
2	541 042 (18.99)	16 554 (17.41)	524 488 (19.05)		3544 (17.55)	55 (16.62)	3489 (17.56)	
3	593 564 (20.84)	16 315 (17.16)	577 249 (20.96)		4144 (20.52)	58 (17.52)	4086 (20.57)	
4	613 369 (21.53)	17 119 (18)	596 250 (21.65)		4811 (23.82)	74 (22.36)	4737 (23.85)	
5 (most deprived)	623 615 (21.89)	22 507 (23.67)	601 108 (21.83)		5061 (25.06)	70 (22.15)	4991 (25.13)	
Unknown	45 559 (1.57)	1227 (1.27)	44 332 (1.49)		276 (1.35)			
Psychiatric history				**<0**.**0001**				0.4058
No	2 574 477 (88.96)	83 224 (86.41)	2 491 253 (89.04)		8970 (43.82)	155 (46.13)	8815 (43.78)	
Yes	319 611 (11.04)	13 089 (13.59)	306 522 (10.96)		11 501 (56.18)	181 (53.87)	11 320 (56.22)	
Duration of reference hospital stay (days) – median (IQ)	3 (1–6)	7 (3–14)	3 (1–6)	**<0**.**0001**	1 (0–3)	6 (2–12)	1 (0–2)	**<0**.**0001**
Level of clinical care				**<0**.**0001**				**0**.**0252**
L1: General hospital ward^a^	2 528 207 (87.36)	72 839 (75.63)	2 455 368 (87.76)		17 175 (83.90)	264 (78.57)	16 911 (83.99)	
L2: ICU without invasive procedure^b^	301 188 (10.41)	13 557 (14.08)	287 631 (10.28)		2268 (11.08)	51 (15.18)	2217 (11.01)	
L3: ICU with invasive procedure^c^	64 693 (2.24)	9917 (10.30)	54 776 (1.96)		1028 (5.02)	21 (6.25)	1007 (5.00)	

ICU, intensive care unit.

a. Level 1 (L1): patients with the mildest level of respiratory difficulty admitted to a general hospital ward (medical, surgery, obstetrics) who required no or low-flow oxygen (up to 15 L/min).

b. Level 2 (L2): patients admitted to an ICU irrespective of the intensity (i.e. type and flowrate) of oxygen supply therapy, and patients who received high-flow nasal oxygen or non-invasive ventilation.

c. Level 3 (L3): patients who were admitted to an ICU and required at least invasive ventilatory support.

Over the 12-month period following discharge from their initial hospital stay, 20 471 (0.71%) individuals were admitted to hospital for self-harm. Of these, 0.35% (*n* = 336) had previously been admitted to hospital for COVID-19, and 0.72% (*n* = 20 135) for another reason.

The gender ratio was not significantly different in the two groups (i.e. COVID-19 versus other reason) (*P* = 0.5807) (Table 1). Those initially admitted to hospital for COVID-19 were more likely to belong to the two older age groups (i.e. 70–74, 75+) (*P* < 0.0001). Patients initially admitted to hospital for reasons other than COVID-19 had a slightly higher (i.e. poorer) deprivation index score (*P* < 0.0001). The two groups did not differ in terms of psychiatric history (*P* = 0.4058). A significant difference was observed for the duration of initial stay in hospital: 6 days for COVID-19 patients *v.* 1 day for patients admitted to hospital for other reasons (*P* < 0.0001). Finally, a greater proportion of COVID-19 patients were admitted to ICU (i.e. levels 2 and 3 of care) (*P* = 0.0252).

### Risk of admission to hospital for self-harm according to variables of interest

Associations between admission for self-harm during the 12 months following discharge from hospital for COVID-19 or for another reason and sociodemographic factors, psychiatric disorder history and characteristics of the initial admission are shown in Table 2.

**Table 2:** Odds ratio, adjusted odds ratio (aOR) and 95% CI (in bold when statistically significant) for the risk of admission to hospital for self-harm in the 12 months following hospital discharge for patients admitted for COVID-19 versus those admitted for another reason (in all adult patients admitted in metropolitan France in the first half of 2020)

Characteristic	Model 1^a^	Model 2^b^	Model 3^c^	Model 4^d^
	Odds ratio (95% CI)	aOR (95% CI)	aOR (95% CI)	aOR (95% CI)
Admission for COVID-19				
No	1	1	1	1
Yes	**0.52 [0.46–0.59]**	**0.66 [0.59–0.73]**	**0.65 [0.58–0.73]**	**0.70 [0.63–0.78]**
Gender				
Male		1	1	1
Female		**0.84 [0.81–0.86]**	**0.84 [0.81–0.86]**	**0.88 [0.85–0.91]**
Age (years)				
18–39		1	1	1
40–59		**1.05 [1.01–1.08]**	**0.73 [0.71–0.76]**	**0.73 [0.71–0.76]**
60–74		**0.28 [0.27–0.29]**	**0.23 [0.22–0.24]**	**0.24 [0.23–0.25]**
75+		**0.17 [0.16–0.18]**	**0.12 [0.12–0.13]**	**0.15 [0.14–0.15]**
Social deprivation index (quintiles*)*				
1 (least deprived)		1	1	1
2		0.98 [0.93–1.04]	0.99 [0.94–1.04]	0.99 [0.94–1.04]
3		**1.07 [1.02–1.13]**	1.02 [0.97–1.08]	1.03 [0.98–1.09]
4		**1.18 [1.12–1.24]**	**1.12 [1.06–1.18]**	**1.13 [1.08–1.19]**
5 (most deprived)		**1.16 [1.10–1.22]**	**1.08 [1.02–1.13]**	**1.10 [1.04–1.15]**
Psychiatric disorder history				
No			1	1
Yes			**12.03 [11.69–12.38]**	**11.91 [11.57–12.26]**
Duration of initial hospital stay (days) – mean (s.d.)				1
Level of clinical care received				**0.93 [0.93–0.94]**
Level 1: General hospital ward^a^				1
Level 2: ICU without invasive procedure^b^				**1.76 [1.68–1.80]**
Level 3: ICU with invasive procedure^c^				**4.19 [3.91–4.50]**

ICU, intensive care unit.

a. No adjustment.

b. Odds ratio adjusted for sociodemographic characteristics: gender, age, region of residence and social deprivation index.

c. Odds ratio adjusted for sociodemographic characteristics and history of psychiatric disorder.

d. Odds ratio adjusted for sociodemographic characteristics, history of psychiatric disorder and characteristics of initial admission (i.e. duration of hospital stay (days) and level of clinical care received).

e.Level 1: patients with the mildest level of respiratory difficulty admitted to a general hospital ward (medical, surgery, obstetrics) who required no or low-flow oxygen (up to 15 L/min).

f.Level 2: patients admitted to an ICU irrespective of the intensity (i.e. type and flowrate) of oxygen supply therapy, and patients who received high-flow nasal oxygen or non-invasive ventilation.

g.Level 3: patients who were admitted to an ICU and required at least invasive ventilatory support.

A negative association was found between initial admission to hospital for COVID-19 and subsequent admission to hospital for self-harm (Model 1: odds ratio = 0.52, 95% CI: 0.46–0.59, *P* < 0.0001). This association remained significant but was attenuated after adjusting for sociodemographic factors (Model 2: (aOR) = 0.66, 95% CI: 0.59–0.73, *P* < 0.0001), for psychiatric disorder history (Model 3: aOR = 0.65, 95% CI: 0.58–0.73, *P* < 0.0001) and for the characteristics of the initial admission to hospital (Model 4: aOR = 0.70, 95% CI: 0.63–0.78, *P* < 0.0001).

In the final model (Model 4), psychiatric disorder history was by far the variable most associated with admission to hospital for self-harm (aOR = 11.91, 95% CI: 11.57–12.26, *P* < 0.0001). Patient age and the level of clinical care received during initial admission to hospital were also associated with it. Specifically, those aged 75+ years were much less likely to be admitted to hospital than the 18–39 age group (aOR = 0.15, 95% CI: 0.14–0.15, *P* = 0.0006). When the social deprivation index score was high (4 and 5), the association with the outcome was stronger (e.g. level 5/1, aOR = 1.10, 95% CI: 1.04–1.15, *P* < 0.00051). Those who received level 2 (aOR = 1.76, 95% CI: 1.68–1.80, *P* < 0.0001) and/or level 3 (aOR = 4.19, 95% CI: 3.91–4.50, *P* < 0.0001) care were at higher risk of admission to hospital than those who received only level 1 care (see above).

### Stratified analyses

After stratification by gender, multivariable analyses showed a strengthened similar negative association for the risk of subsequent admission to hospital for suicide attempts in both male and female patients (Model 5: aOR = 0.75, 95% CI: 0.64–0.87 and aOR = 0.63, 95% CI: 0.53–0.74, respectively) (Supplementary Table 1 available at https://doi.org/10.1192/bjo.2024.786). In the same way, multivariable analyses stratified by age showed the same pattern, except for the 75+ category, as well as for those stratified by level of intensity of clinical care (Table 3).

**Table 3:** Adjusted odds ratio (aOR) and 95% CI (in bold when statistically significant) for the risk of subsequent admission to hospital for suicide attempts over the 12-month period after initial hospital discharge, for patients admitted for COVID-19 versus those admitted for another reason, in all adult patients admitted in metropolitan France the first half of 2020: Model 5, stratified by age categories (18–39, 40–59, 60–74 and 75+) using model adjusted for all variables (Model 4)

Characteristic	18–39	40–59	60–74	75 and more
	aOR (95% CI)	aOR (95% CI)	aOR (95% CI)	aOR (95% CI)
Model 4				
Admission for COVID-19				
No	1	1	1	1
Yes	**0.61 [0.47–0.79]**	**0.62 [0.51–0.74]**	**0.55 [0.42–0.73]**	1.05 [0.85–1.31**]**
Gender				
Male	1	1	1	1
Female	**0.64 [0.61–0.68]**	**1.11 [1.06–1.16]**	**1.41 [1.31–1.53]**	1.03 [0.94–1.14]
Social deprivation index (quintiles)				
1 (least deprived)	1	1	1	1
2	0.96 [0.88–1.05]	1.04 [0.95–1.13]	0.92 [0.80–1.06]	0.98 [0.83–1.17]
3	1.07 [0.98–1.17]	1.03 [0.95–1.13]	0.99 [0.86–1.14]	0.96 [0.81–1.14]
4	**1.13 [1.04–1.23]**	**1.19 [1.10–1.30]**	1.08 [0.94–1.23]	1.00 [0.84–1.18]
5 (most deprived)	**1.09 [1.00–1.19]**	**1.13 [1.04–1.23]**	1.00 [0.87–1.15]	1.10 [0.93–1.29]
Psychiatric disorder history				
No	1	1	1	1
Yes	**14.22 [13.57–14.91]**	**10.96 [10.46–11.48]**	**11.31 [10.46–12.24]**	**3.90 [3.53–4.30]**
Duration of initial hospital stay (days) – mean (s.d.)	1	1	1	1
Level of clinical care received	**0.85 [0.84–0.86]**	**0.93 [0.92–0.93]**	**0.97 [0.97–0.98]**	**0.99 [0.98–0.99]**
Level 1: General hospital ward^a^	1	1	1	1
Level 2: ICU without invasive procedure^b^	**3.02 [2.78–3.28]**	**1.45 [1.35–1.57]**	**1.27 [1.14–1.43]**	**1.33 [1.17–1.53]**
Level 3: ICU with invasive procedure^c^	**7.62 [6.68–8.69]**	**3.92 [3.52–4.37]**	**2.74 [2.32–3.24]**	**1.99 [1.48–2.69]**

ICU, intensive care unit

Model 4: odds ratio adjusted for sociodemographic characteristics, history of psychiatric disorder and characteristics of initial admission (i.e. duration of hospital stay (days) and level of clinical care received).

a. Level 1: patients with the mildest level of respiratory difficulty admitted to a general hospital ward (medical, surgery, obstetrics) who required no or low-flow oxygen (up to 15 L/min).

b. Level 2: patients admitted to an intensive care unit (ICU) irrespective of the intensity (i.e. type and flowrate) of oxygen supply therapy, and patients who received high-flow nasal oxygen or non-invasive ventilation.

c. Level 3: patients who were admitted to an ICU and required at least invasive ventilatory support.

### Sensitivity analyses

Our two sensitivity analyses, one based on a more restricted initial admission to hospital period (16 March–30 June 2020) and the other excluding initial admission to hospital for psychiatric reasons, showed similar results (Supplementary Tables 2 and 3).

## Discussion

### Associations

Our aim was to compare the risk of admission to hospital for self-harm in the 12 months following staying in hospital during the first half of 2020 for COVID-19 with the risk following admission to hospital for other reasons. We have highlighted that patients who had previously been admitted to hospital for COVID-19 had a lower risk of being admitted to hospital for self-harm in the 12 months following discharge than those previously admitted to hospital for other reasons, including after successive adjustment, and in stratified analyses. This result contrasts with findings in another study on the same population, where we found a higher risk of admission to hospital for other psychiatric disorders (respectively a psychiatric disorder of any type, psychotic and anxiety disorders) in the 12 months following discharge from hospital for COVID-19 than following discharge for another reason.^4^ In our former study, this risk remained significantly higher even after adjustment for sociodemographic data and psychiatric disorder history.^4^

The contrast between our previous and present work echoes contrasts observed elsewhere between the ‘admission to hospital for self-harm’ indicator and other indicators of mental health status in the French context. For example, although a previous national survey showed high levels of depression, anxiety, insomnia and suicidal ideation in the general population in the COVID-19 period,^23^ studies of hospital admissions for self-harm showed a global downward trend during the same period.^24^ Studies elsewhere have also provided contrasting results. Santomauro et al^25^ suggested that the COVID-19 health crisis is associated with a significant impact on mental health worldwide, while other studies indicated lower admission to hospital rates for self-harm in 2020–2021 than in the preceding years.^11,26^ This contrast might be partly because most of the studies published to date on the status of mental health in general populations during the COVID-19 pandemic used self-reported internet-based questionnaires and self-reporting is likely to be biased toward those mostly emotionally affected. Another possible reason is the failure to seek treatment after a self-harm attempt; according to a previous French study, as much as 40% of those who self-harm do not subsequently go to a hospital.^24^ During the first months of the COVID-19 crisis, the rate of admission to hospital for self-harm (and for psychiatric disorders in general) substantially decreased in France in a context of reorganisation of hospital services with restrictions on new admissions and closure of daily care.^13^

In our study, the lower rate of hospital admissions for self-harm in patients who had previously been admitted for COVID-19 than in patients previously admitted for other reasons could be partly explained by the fact that the former may have felt part of what has been termed a ‘pulling together’ phenomenon.^27^ Accordingly, COVID-19 survivors were less likely to feel excluded and more likely to receive both stronger support from their family and better follow-up from healthcare teams following discharge. Such a situation would have most probably lowered the risk of self-harm. A second explanation would be the existence of a ‘post-COVID honeymoon’ during which, as in the early periods of a natural disaster,^28^ the survivor would display a positive affect and feel general relief, which in turn may have reduced suicidality.^29^ A third explanation for the observed discrepancy is that those who were admitted to hospital for reasons other than COVID-19 during the study period may have had diseases that were more severe or chronic in nature, which may have led to a higher risk of self-harm.^16^ Reduced access to care at the start of the pandemic may have led to a selection of more severe cases in our control group ‘admitted to hospital for other reasons than COVID-19’; lower admission rates for all other illnesses were found during this period.^30^ Future research should investigate this further by detailing the ‘other’ group.

A fourth possible reason that we cannot exclude is the possibility that more people admitted to hospital for COVID-19 died during the follow-up period than those admitted to hospital for other reasons (however, death certificates are still unavailable for the period of follow-up of our study). In support of this hypothesis, a study carried out in England on health data recorded by the National Health Service seems to show an excess of all-cause mortality in the months following admission to hospital for COVID-19 compared with admission to hospital for influenza.^31^

In our study, admission to hospital for self-harm decreased progressively with increasing age. This result is consistent with previous data highlighting that admission rates were highest in adolescent girls followed by middle-aged persons, with a clear decrease being observed in elderly patients.^32^ Moreover, a previous study conducted in France found that the rate of admission to hospital for self-harm from the summer of 2020 onwards was higher for adolescents and young adults than in previous years, while the rate for middle-aged people decreased and the rate for people aged 70 and over remained unchanged.^33^ Furthermore, Fernando et al.^16^ showed among those admitted to an ICU a protective effect regarding a risk of consecutive self-harm of (a) being older and (b) receiving long-term care in a medical facility after discharge. In our study, patients in the COVID-19 group were significantly older than those in the ‘other reasons’ group. They were therefore more likely to have been referred to a long-term care facility or to a nursing home upon discharge. In addition, the health crisis may have led to a reorganisation of care in favour of COVID-infected patients, who received priority care after admission to hospital, which may have reduced the risk of self-harm.

We also found a significant association between the risk of admission to hospital for self-harm and the level of social deprivation; this finding is consistent with the literature.^34^ Finally, our models highlighted a lower risk of self-harm in admitted women. This contrasts with findings in the literature on the general population.^35^

A history of psychiatric disorder was strongly associated with the risk of admission to hospital for self-harm in our sample, also reflecting the literature.^16^

We found a strong association between the level of care received in initial admission to hospital and the subsequent risk of admission to hospital for self-harm, reflecting recent findings elsewhere;^16^ this is an important result in a context where intensive care and mental health suffering are now common because of the COVID-19 crisis.

### Strengths and limitations

Our study has several strengths. First, the SNDS health database provides complete data on almost the entire French population.^4^ This ensured sufficient statistical power to analyse the risk of admission to hospital for self-harm after previous admission to hospital for COVID-19 versus admission to hospital for another reason. Moreover, using SNDS data made it possible to avoid recall, declaration and selection biases, elements that are particularly relevant when one considers that the issue of self-harm is still very much taboo.

Second, the choice to study admissions to hospital for COVID-19 with admissions to hospital for other reasons during the first wave in France allowed us to take into account this unique period in the pandemic, which was characterised by a stringent lockdown, strict isolation and generalised concern about infection. However, this exceptional period is not representative of the entire health crisis linked to COVID-19, as it gave rise to major changes in the organisation of care and reduced access to care.

Third, comparing patients admitted to hospital for COVID-19 to other admitted patients enabled us to take into account the vulnerability related to admittance to hospital itself, and to analyse the specific effect of COVID-19.

The study also has limitations. First, although the national sample made it possible to study self-harm after admission to hospital for COVID-19 or for another reason, the actual number of admissions for self-harm was relatively small. For this reason, our results require careful replication and interpretation. Second, our study is based only on self-harm that led to admission to hospital. Persons who self-harmed but received no or only outpatient psychiatric care were not considered; this limits the generalisability of our data. Indeed, only a portion of persons who self-harm in France receive inpatient care.^26^ This was particularly true during the height of the COVID-19 health crisis when there was a lack of inpatient beds.^13^ Furthermore, admission to hospital for ‘self-harm’ does not inform us about suicidal intent as such; further studies should address this point using other databases with a more detailed clinical description, distinguishing suicide attempt and non-suicidal self-harm.

Third, a temporary change in coding practices during the COVID-19 crisis cannot be ruled out, as admission to hospital for self-harm was coded as an associated diagnosis. However, there is no obvious reason that might explain how this hypothetical change would have a differential effect on the number of registered admissions to hospital for self-harm in the COVID-19 group versus the ‘other reasons’ group.

Fourth, we considered the 12 months following discharge after admission to hospital for COVID-19 or for another reason. Although this is a longer period than the majority of studies looking at the psychiatric consequences of COVID-19,^36^ it may still be too short: data on long COVID or post-COVID conditions show that the long-term consequences of COVID-19 might appear even later, particularly in cases of psychiatric history.^37^ In terms of suicide, a 12-year follow-up of a cohort of individuals infected by the 2003 SARS epidemic previously showed increased rates of suicide several years after infection.^38^ Accordingly, a longer-term impact of COVID-19 infection on self-harm cannot be ruled out, either because of persistent biological phenomena or because of psychosocial and economic factors following the related crisis.^39^ Clinicians and researchers need to follow patients over the long term to better understand the disease's impact on suicide risk with a view to preventing attempts.

Finally, other studies should focus on patients under 18 years of age, as they are particularly prone to self-harm.^40^

To our knowledge, this is the first study to investigate the risk of admission to hospital for self-harm in the 12 months following discharge after admission to hospital for COVID-19 or for another reason using data from the French administrative healthcare database. Our findings indicate that initial admission to hospital for COVID-19 (versus another reason) was significantly associated with a lower risk of admission to hospital for self-harm. Our study highlights the importance of taking into account psychiatric disorder history and intensive care management (especially as they are frequent in the ongoing COVID-19 crisis) when evaluating the risk of subsequent self-harm. Finally, not enough time has elapsed since the beginning of the pandemic to have a complete picture of all the long-term possible consequences of COVID-19. Longer-term follow-up of patients and further studies are therefore needed to fully understand and guide future health policy.

## Supporting information

Pirard et al. supplementary material Pirard et al. supplementary material

## Data Availability

The national French administrative healthcare database, Système National des Données de Santé (SNDS [National Administrative Healthcare Database]), comprises a set of strictly pseudonymised and protected databases. Access to individual data in these systems for research purposes is only possible in the SNDS hub and data cannot be extracted or shared.
