# Supplementary material for: Risk of admission to hospital for self-harm after admission to hospital for COVID-19: French nationwide longitudinal study
Source: BJPsych Open. 2024 Dec 5;10(6):e215. doi: 10.1192/bjo.2024.786 (PMC11698173; doi:10.1192/bjo.2024.786)
Supplement: Pirard et al. supplementary material [file S2056472424007865sup001.docx]

**Table S1.** Adjusted Odds ratio (OR) and 95% confidence interval (95%CI) for the risk of subsequent hospitalization for suicide attempts over the 12-months period after initial hospital discharge, for patients hospitalized for COVID-19 versus those hospitalized for another reason, in all adult patients hospitalized in metropolitan France the first half of 2020: Model 5, stratified by sex using model adjusted for all variables (Model 4) and Model 6, stratified by level of clinical care using model adjusted for all variables (Model 4)

|  |  | *Hospitalization for COVID-19 vs hospitalization for another reason* |
| --- | --- | --- |
|  |  | aOR (95%CI) π |
| Model 5 |  |  |
|  | Male | **0.75 [0.64 -0.87]** |
|  | Female | **0.63 [0.53-0.74]** |
| Model 6 |  |  |
|  | ^1^ Level 1: General hospital ward | **0.84 [0.74-0.96]** |
|  | ^2^ Level 2: Intensive care unit | **0.61 [0.46-0.80]** |
|  | ^3^ Level 3: Intensive care unit with invasive procedures | **0.22 [0.14-0.34]** |

|  | Model 1 ^†^ | Model 2 ^‡^ | Model 3 ^§^ | Model 4 ^π^ |
| --- | --- | --- | --- | --- |
|  | OR (95%CI) | aOR (95%CI) | aOR (95%CI) | aOR (95%CI) |
| *hospitalization for COVID-19* |  |  |  |  |
| No | 1 | 1 | 1 | 1 |
| Yes | 0.45 [0.40-0.50] | 0.60 [0.54-0.67] | 0.58 [0.52-0.66] | 0.63 [0.56-0.71] |
| *Sex* |  |  |  |  |
| Male |  | 1 | 1 | 1 |
| Female |  | 0.83 [0.79-0.86] | 0.82 [0.79-0.85] | 0.87 [0.83-0.90] |
| *Age (years)* |  |  |  |  |
| 18-39 |  | 1 | 1 | 1 |
| 40-59 |  | 1.18 [1.13-1.24] | 0.82 [0.79-0.86] | 0.82 [0.78-0.86] |
| 60-74 |  | 0.33 [0.31-0.35] | 0.26 [0.25-0.28] | 0.27 [0.26-0.29] |
| 75+ |  | 0.20 [0.18-0.21] | 0.14 [0.13-0.15] | 0.17 [0.16-0.18] |
| *Social deprivation index (quintiles)* |  |  |  |  |
| 1 (least deprived) |  | 1 | 1 | 1 |
| 2 |  | 0.99 [0.93-1.07] | 0.99 [0.92-1.07] | 0.99 [0.92-1.07] |
| 3 |  | 1.02 [0.95-1.10] | 0.97 [0.90-1.04] | 0.98 [0.91-1.05] |
| 4 |  | 1.18 [1.10-1.26] | 1.11 [1.03-1.19] | 1.12 [1.05-1.21] |
| 5 (most deprived) |  | 1.14 [1.06-1.22] | 1.05 [0.98-1.13] | 1.07 [1.00-1.15] |
| *Psychiatric disorder history* |  |  |  |  |
| No |  |  | 1 | 1 |
| Yes |  |  | 11.40 [10.95-11.87] | 11.22 [10.77-11.68] |
| *Duration of initial hospital stay (days) - mean(SD)* |  |  |  | 1 |
| *Level of clinical care received* |  |  |  | 0.94 [0.93-0.94] |
| ^1^ Level 1: General hospital ward |  |  |  | 1 |
| ^2^ Level 2: ICU without invasive proc. |  |  |  | 1.72 [1.61-1.83] |
| ^3^ Level 3: ICU with invasive procedure |  |  |  | 4.14 [3.77-4.55] |

**Table S2.**  Odds-ratio (OR), adjusted odds-ratio (aOR), and 95% confidence intervals (95%CI) for the risk of hospitalization for self-harm in the 12 months following hospital discharge for patients hospitalized for COVID-19 versus those hospitalized for another reason (in all adult patients hospitalized in metropolitan France ***between 16 March 2020 and 30 June 2020***).

| † No adjustment |
| --- |
| ‡ *Odds-ratio* adjusted for socio-demographic characteristics: sex, age, region of residence, and social deprivation index |
| *§ Odds-ratio* adjusted for socio-demographic characteristics and history of psychiatric disorder  ^1^ Level 1: patients with the mildest level of respiratory difficulty admitted to a general hospital ward (medical, surgery, obstetrics) who required no or low-flow oxygen (up to 15 L/min).  ^2^ Level 2: patients admitted to an intensive care unit (ICU) irrespective of the intensity (i.e., type and flowrate) of oxygen supply therapy, and patients who received high-flow nasal oxygen or non-invasive ventilation;  ^3^ Level 3: patients who were hospitalized in an ICU and required at least invasive ventilatory support |
|  |

|  | Model 1 ^†^ | Model 2 ^‡^ | Model 3 ^§^ | Model 4 ^π^ |
| --- | --- | --- | --- | --- |
|  | OR (95%CI) | aOR (95%CI) | aOR (95%CI) | aOR (95%CI) |
| *hospitalization for COVID-19* |  |  |  |  |
| No | 1 | 1 | 1 | 1 |
| Yes | **0.48 [0.42-0.53]** | **0.64 [0.57-0.71]** | **0.60 [0.54-0.67]** | **0.66 [0.59-0.74]** |
| *Sex* |  |  |  |  |
| Male |  | 1 | 1 | 1 |
| Female |  | **0.81 [0.79-0.84]** | **0.78 [10.76-0.81]** | **0.82 [0.80-0.84]** |
| *Age (years)* |  |  |  |  |
| 18-39 |  | 1 | 1 | 1 |
| 40-59 |  | **1.05 [1.01-1.081]** | **0.71 [0.69-0.73]** | **0.71 [0.69-0.74]** |
| 60-74 |  | **0.27 [0.26-0.28]** | **0.21 [0.20-0.22]** | **0.22 [0.21-0.23]** |
| 75+ |  | **0.16 [0.16-0.17]** | **0.11 [0.11-0.12]** | **0.13 [0.13-0.14]** |
| *Social deprivation index (quintiles)* |  |  |  |  |
| 1 (least deprived) |  | 1 | 1 | 1 |
| 2 |  | 0.98 [0.93-1.04] | 0.99 [0.93-1.04] | 0.99 [0.94-1.04] |
| 3 |  | **1.08 [1.02-1.13]** | 1.02 [0.97-1.08] | 1.03 [0.98-1.09] |
| 4 |  | **1.19 [1.13-1.25]** | **1.11 [1.06-1.17]** | **1.13 [1.07-1.19]** |
| 5 (most deprived) |  | **1.17 [1.11-1.23]** | **1.07 [1.02-1.13]** | **1.09 [1.04-1.15]** |
| *Psychiatric disorder history* |  |  |  |  |
| No |  |  | 1 | 1 |
| Yes |  |  | **13.78 [13.83-14.18]** | **13.67 [13.28-14.0.8]** |
| *Duration of initial hospital stay (days) - mean(SD)* |  |  |  | 1 |
| *Level of clinical care received* |  |  |  | **0.93 [0.92-0.93**] |
| ^1^ Level 1: General hospital ward |  |  |  | 1 |
| ^2^ Level 2: ICU without invasive proc. |  |  |  | **1.63 [1.56-1.71]** |
| ^3^ Level 3: ICU with invasive proc. |  |  |  | **3.95 [3.68-4.24]** |

| **Table S3** Odds-ratio (OR), adjusted odds-ratio (aOR), and 95% confidence intervals (95%CI) for the risk of hospitalization for self-harm in the 12 months following hospital discharge for patients hospitalized for COVID-19 versus those hospitalized for another **reason (excluding for psychiatric disorders)** (in all adult patients hospitalized in metropolitan France in the first half of 2020). |
| --- |
|  |

| † No adjustment |
| --- |
| ‡ *Odds-ratio* adjusted for socio-demographic characteristics: sex, age, region of residence, and social deprivation index |
| *§ Odds-ratio* adjusted for socio-demographic characteristics and history of psychiatric disorder  ^1^ Level 1: patients with the mildest level of respiratory difficulty admitted to a general hospital ward (medical, surgery, obstetrics) who required no or low-flow oxygen (up to 15 L/min).  ^2^ Level 2: patients admitted to an intensive care unit (ICU) irrespective of the intensity (i.e., type and flowrate) of oxygen supply therapy, and patients who received high-flow nasal oxygen or non-invasive ventilation;  ^3^ Level 3: patients who were hospitalized in an ICU and required at least invasive ventilatory support |
